# Pseudoaneurysm of the Anterior Tibial Artery following Ankle Arthroscopy in a Soccer Player

**DOI:** 10.1155/2017/2865971

**Published:** 2017-05-18

**Authors:** Ichiro Tonogai, Tetsuya Matsuura, Toshiyuki Iwame, Keizo Wada, Tomoya Takasago, Tomohiro Goto, Daisuke Hamada, Yohei Kawatani, Eiki Fujimoto, Tetsuya Kitagawa, Shyoichiro Takao, Seiji Iwamoto, Moriaki Yamanaka, Masafumi Harada, Koichi Sairyo

**Affiliations:** ^1^Department of Orthopedics, Institute of Biomedical Science, Tokushima University Graduate School, Tokushima, Japan; ^2^Department of Cardiovascular Surgery, Institute of Biomedical Science, Tokushima University Graduate School, Tokushima, Japan; ^3^Department of Radiology, Institute of Biomedical Science, Tokushima University Graduate School, 3-18-15 Kuramoto, Tokushima 770-8503, Japan

## Abstract

Ankle arthroscopy carries a lower risk of vascular complications when standard anterolateral and anteromedial portals are used. However, the thickness of the fat pad at the anterior ankle affords little protection for the thin-walled anterior tibial artery, rendering it susceptible to indirect damage during procedures performed on the anterior ankle joint. To our knowledge, only 11 cases of pseudoaneurysm involving the anterior tibial artery after ankle arthroscopy have been described in the literature. Here we reported a rare case of a 19-year-old soccer player who presented with pseudoaneurysm of the anterior tibial artery following ankle arthroscopy using an ankle distraction method and underwent anastomosis for the anterior tibial artery injury. Excessive distraction of the ankle puts the neurovascular structures at greater risk for iatrogenic injury of the anterior tibial artery during ankle arthroscopy. Surgeons should look carefully for postoperative ankle swelling and pain after ankle arthroscopy.

## 1. Introduction

Arthroscopy of the ankle was first described in 1939 by Takagi [1] and since the 1970s has become an important diagnostic and therapeutic tool for the trauma and orthopedic surgeon. Ankle arthroscopy has well established benefits and is considered to carry a relatively low risk of vascular injury, especially when anterolateral and anteromedial portals are used. However, there are definite risks associated with the procedure, and reported complication rates range from 9% [2] to 17% [3]. Most of the complications are neurological but vascular injuries also occur.

A pseudoaneurysm (or false aneurysm) can occur when there is injury to all three layers of an artery, resulting in extravasation of blood and formation of a fibrous capsule containing blood flowing outside the lumen of the damaged vessel. The incidence of pseudoaneurysm after arthroscopy has been reported to be 0.008% [4] and most cases have involved the popliteal vessels after knee arthroplasty [5–8]. Pseudoaneurysms of the foot and ankle are rare but can be caused by an ankle sprain, ankle fracture, fixation of a fracture, or ankle arthroscopy. The anterior tibial artery is the vessel most commonly involved in cases of pseudoaneurysm of the foot and ankle [9].

To our knowledge, only 11 cases of pseudoaneurysm involving the anterior tibial artery after ankle arthroscopy have been described in the literature. In this paper, we describe a rare case of a 19-year-old soccer player who presented with a pseudoaneurysm of the anterior tibial artery following ankle arthroscopy with debridement of anterior tibiotalar osteophytes and synovectomy using standard anteromedial and anterolateral portals, in whom an anastomosis was performed to repair the anterior tibial artery injury.

## 2. Case Report

**Figure 1:**
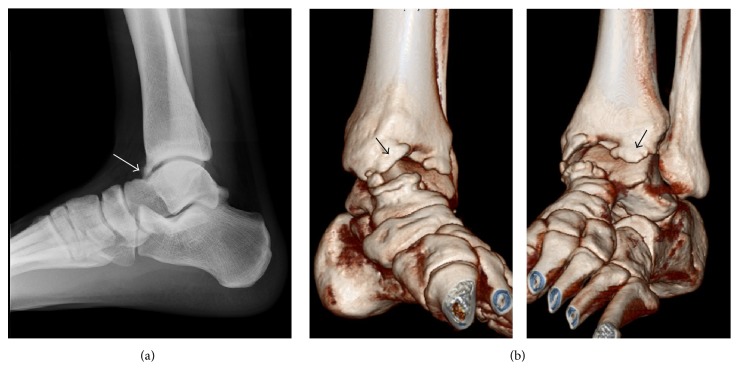
(a) Plain radiograph and (b) three-dimensional computed tomography scan acquired at initial consultation indicating osteophytes at the anterior edge of the distal tibial end and the dorsal side of the talar neck ((a) white arrow, (b) black arrow).

An otherwise healthy 19-year-old male soccer player experienced left ankle pain when playing soccer at a competitive university level. The pain was severe enough that he could not continue with practice, so he consulted a local doctor the following day. A plain radiograph and three-dimensional computed tomography scan demonstrated osteophytes at the anterior edge of the distal end of the tibia and dorsal talar neck, indicating anterior ankle impingement syndrome (Figures 1(a) and 1(b)). The patient was referred to our department 2 weeks later. On physical examination, there was tenderness over the anteromedial aspect of the ankle and range of motion at the ankle joint was limited to 5° of dorsiflexion.

**Figure 2:**
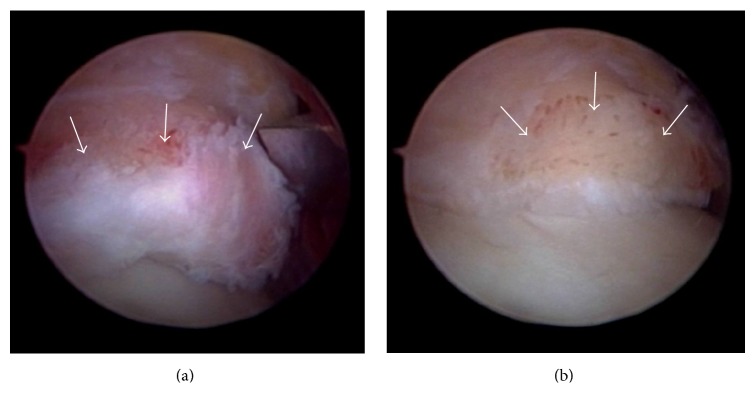
Photographs taken during ankle arthroscopy showing (a) large osteophytes at the anterior edge of the distal tibial end (arrow). (b) After excision using a bone cutter (arrow).

**Figure 3:**
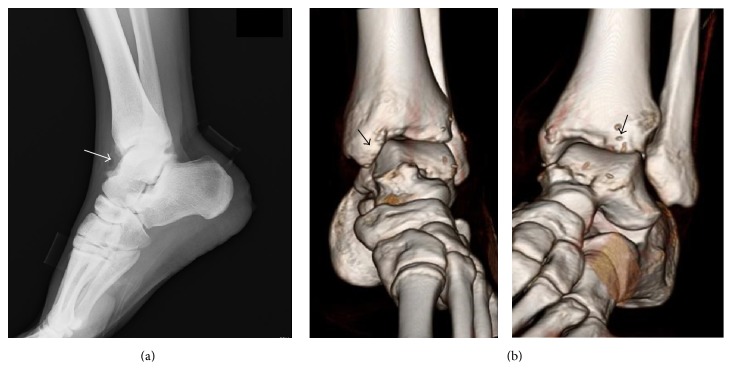
(a) A plain radiograph and (b) a three-dimensional computed tomography scan after ankle arthroscopy showing resection of osteophytes at the anterior edge of the distal tibial end and at the dorsal talar neck ((a) white arrow, (b) black arrow).

Three weeks after the first presentation to us, he underwent arthroscopic debridement of anterior tibiotalar osteophytes and synovectomy for the anterior ankle impingement syndrome. Under general anesthesia and with a tourniquet, noninvasive distraction was applied using an ankle strap and foot traction. An arthroscopic pump was used to distend the joint. After making an initial incision through the skin, a mosquito hemostat was used to introduce the arthroscope into the articular cavity and create standard anteromedial and anterolateral portals. Ankle arthroscopy revealed large osteophytes at the anterior edge of the distal end of the tibia and the dorsal talar neck (Figure 2(a)), which were excised using a bone cutter (Figure 2(b)). The hypertrophic synovium was resected arthroscopically using a 3.5 mm motorized shaver. On further inspection of the ankle, the cartilage was seen to be normal. The joint was irrigated thoroughly and the portal sites were closed with nylon. Resection of the osteophytes was confirmed after ankle arthroscopy by plain radiography and three-dimensional computed tomography (Figures 3(a) and 3(b)). The patient was permitted to bear weight on his left foot as tolerable as possible

**Figure 4:**
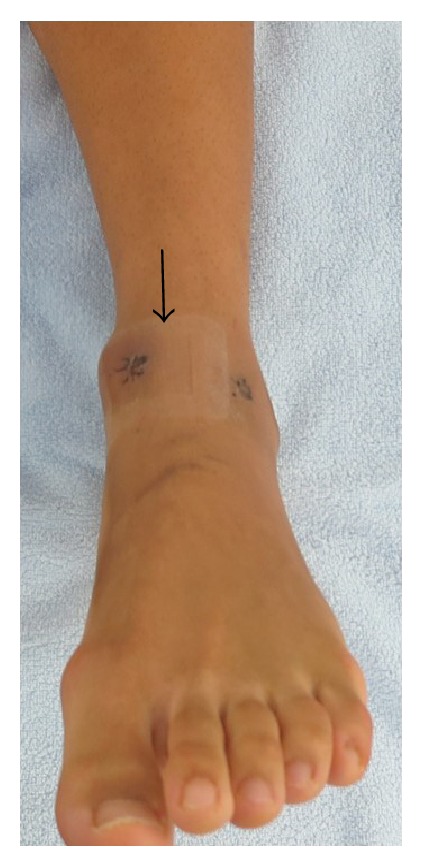
Photograph of the ankle and foot showing swelling between the anteromedial portal and the anterolateral portal (arrow) 3 days after ankle arthroscopy.

The patient experienced no pain in the early postoperative period. After ankle arthroscopy, a normal dorsal artery pulse was palpable. Three days after the ankle arthroscopy, the patient twisted his left leg accidentally, such that his full body weight was suddenly supported by the left foot. Later that day, the patient presented with pain and swelling in the anterior aspect of the ankle (Figure 4), which he considered to be bearable. The sutures were removed 10 days after the ankle arthroscopy.

**Figure 5:**
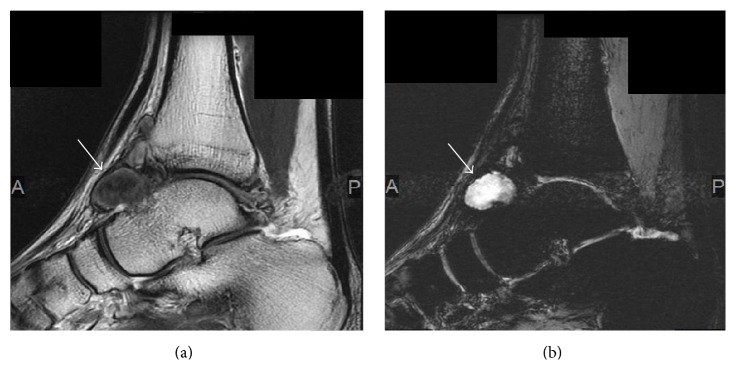
Magnetic resonance images showing a pseudoaneurysm 25 × 22 × 13 mm in size arising from the anterior tibial artery (arrow) at the level of the ankle joint. The pseudoaneurysm is seen as a mass with a heterogeneous (a) low-intensity to isointensity signal on T2-weighted imaging and (b) high-intensity signal on T2 star-weighted imaging.

Twelve days after the arthroscopy, magnetic resonance imaging showed a 25 × 22 × 13 mm pseudoaneurysm with a heterogeneous low-intensity to isointensity signal on T2-weighted imaging (Figure 5(a)) and a high-intensity signal on T2 star-weighted imaging at the level of the anterior ankle joint (Figure 5(b)).

**Figure 6:**
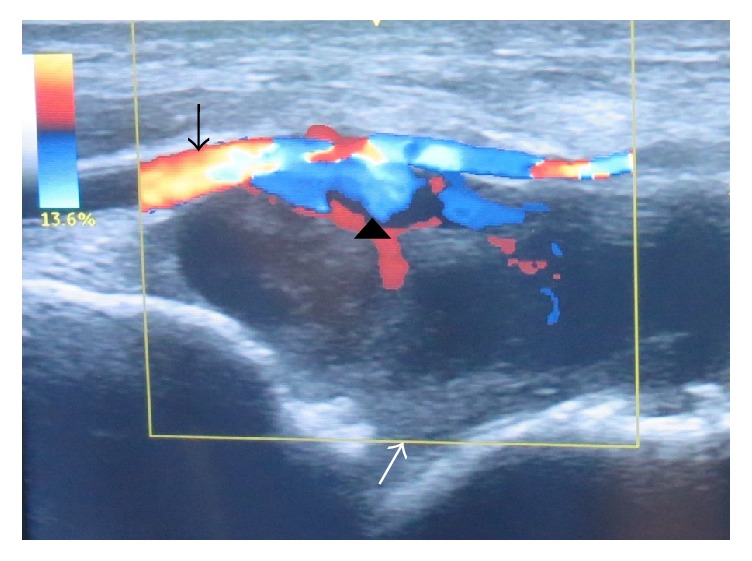
Color and duplex Doppler ultrasonography showing flow through the anterior tibial artery (black arrow) at the anterior ankle joint, with decreased flow through the dorsalis pedis artery, suggesting a pseudoaneurysm (white arrow) leaking into the ankle joint (black arrow head). Flow towards and away from the transducer is indicated by red and blue, respectively.

Thirteen days after arthroscopy, color and duplex Doppler ultrasonographic examination was performed by the radiology team at our hospital. It showed a mosaic pattern of colors with the “whirling blood flow” and “to-and-fro” motion typical of a pseudoaneurysm on the posterior wall of the distal portion of the anterior tibial artery (Figure 6). The final diagnosis was pseudoaneurysm caused by injury of the anterior tibial artery during ankle arthroscopy. The patient was then referred to the cardiovascular surgery team at our hospital. Catheter angiography of the lower limb vasculature was performed to obtain detailed information on the state and integrity of the anterior tibial artery and determine the direction of flow.

**Figure 7:**
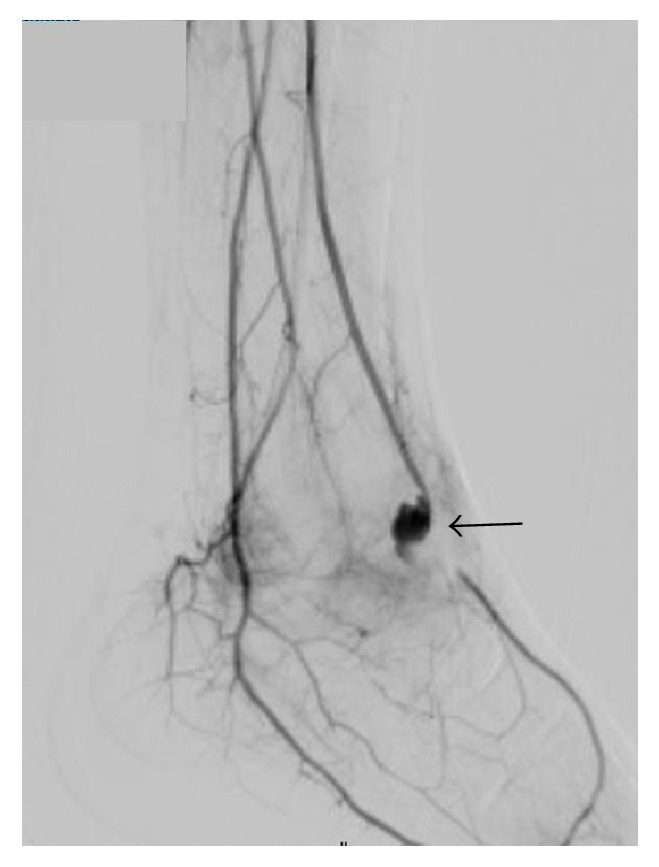
Angiographic image showing a pseudoaneurysm originating from the anterior tibial artery and passing on the anterior side of the ankle joint. Predominantly anterograde filling from the anterior tibial artery is seen (arrow). Flow from the posterior tibial artery and the plantar collateral was also detected. The dorsalis pedis artery is filled by a plantar arch.

**Figure 8:**
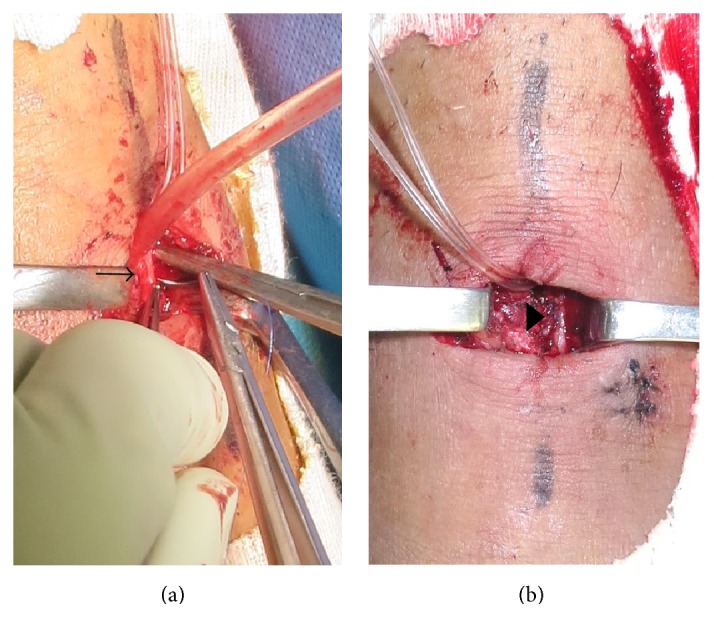
Photographic images taken during repair of the anterior tibial artery injury demonstrated that the pseudoaneurysm was caused by disruption of the posterior third of the anterior tibial artery wall (arrow) on the anterior side of the ankle joint (a). The injured anterior tibial artery wall was repaired using an end-to-end anastomosis longitudinally (arrow head), without compromising the blood supply to the foot (b).

Fourteen days after arthroscopy, angiography was performed by the radiology team at our hospital. It revealed a pseudoaneurysm with an intact posterior tibial artery and plantar arch circulation (Figure 7). Nineteen days after arthroscopy, an anastomosis was created for the injured anterior tibial artery to prevent progression of the ankle pain and swelling. A transverse incision was made over the anterior aspect of the ankle. The pseudoaneurysm was believed to be caused by disruption of the posterior 1/3 of the anterior tibial artery wall (Figure 8(a)) and leakage of blood into the ankle, forming a pseudoaneurysm. The anterior tibial artery was isolated and clipped proximally and distally. The injured arterial walls facing each other were sutured longitudinally with preservation of blood flow distally, although the diameter of the artery became narrower at this point (Figure 8(b)).

Apart from slight paresthesia of the first web space, the patient's subsequent postoperative course was uneventful and he was discharged from hospital 1 week later. The ankle pain and swelling had resolved immediately after repair of the anterior tibial artery injury. He returned to playing soccer at a competitive university level 4 months later.

## 3. Discussion

This paper reports a rare case of a 19-year-old soccer player who underwent anterior tibial artery repair 19 days after ankle arthroscopy for anterior ankle impingement syndrome because magnetic resonance, ultrasonographic, and angiographic imaging performed after the arthroscopy revealed pseudoaneurysm of the anterior tibial artery. A review of the English literature revealed 11 reports of pseudoaneurysm following ankle arthroscopy (see Table 1) [9–20].

Various treatments for a pseudoaneurysm of the anterior tibial artery have been described (Table 1). Surgical ligation or repair of the artery has traditionally been the preferred treatment. Nonsurgical methods described include external compression [21], ultrasound-guided compression [15], ultrasound-guided thrombin injection [22] percutaneous endovascular coil embolization [23, 24], and percutaneous endovascular stenting [25]. The surgical methods described include ligation with aneurysmectomy [12, 13] and repair/reconstruction by either end-to-end anastomosis [26, 27] or grafting [14]. In this case, we opted for surgical treatment because of the patient's strong desire for an early return to playing soccer.

Ligation of the anterior tibial artery can be performed without ischemic complications if the plantar arch is intact and had a collateral blood supply [28–30]. Some authors have postulated that preserving the normal anatomic blood flow with reconstruction might be of benefit in later life if peripheral vascular disease develops [26, 27]. In this case, we chose anastomosis because the patient was young and 1/3 of the posterior wall of the anterior tibial artery was disrupted.

Potential risk factor is the close anatomical relationship of the arteries with the anterior ankle joint capsule [12]. Anatomically, the anterior tibial artery is near the anterior ankle joint capsule at the level of the talar neck and runs deep down the superior and inferior retinaculum. The anterior tibial artery may be located as close as 2.3 mm to the anterior joint capsule [31]. Gentile et al. reported that the distances from the anterior border of the inferior tibial articular facet to the posterior border of the anterior tibial artery were 0.9 cm and 0.7 cm in ankle dorsiflexion and distraction, respectively [32]. Although the cause of pseudoaneurysm in our patient is not clear, the mechanism of injury of the vessel wall may have involved iatrogenic injury by excessive distraction of the ankle during removal of inflamed synovium using a shaver or resection of the osteophytes. Another possibility is strap placement during joint distraction, which can compress the tibial artery in the vicinity of the anterior ankle capsule. Because placement of an ankle strap during joint distraction and placement of the foot in plantar flexion during arthroscopy decreases the distance between the anterior tibial artery and the anterior ankle capsule [15], releasing the distraction force on the ankle may help to decrease the tension on the anterior capsule, thereby decreasing the risk of pseudoaneurysm during debridement of an anterior tibiotalar osteophyte. The distraction method might be advantageous in terms of allowing direct access to the cartilage and the talar dome.

Other potential risk factors for vascular injury include anterior port placement and variability in the anatomic position of the vessel [12]. Lateral and medial deviation of the anterior tibial artery is present in up to 5.5% and 3.5% of the population, respectively [33, 34]. Moreover, Son et al. reported that the anterior tibial artery was located lateral to the extensor digitorum longus and the posterior tibial tendon in 2.0% of subjects, and branching of the artery was observed lateral to these structures in 4.2% of the subjects even though the artery was in the normal position [35]. We do not think that incorrect port positioning contributed to this complication in our patient because no abnormalities in arterial anatomy were seen on angiography or during surgical repair of the anterior tibial artery.

The most severe complication of an untreated pseudoaneurysm of the anterior tibial artery is rupture of this important vessel, given that the fibrous capsule of a pseudoaneurysm is devoid of the natural three-layered architecture of a true aneurysm and expands until it is confined by the limits of adjacent structures. This can be problematic, leading to hemorrhage into the soft tissue and hemodynamic instability, hemarthrosis of the ankle, and compartment syndrome in severe cases [17]. Even though pseudoaneurysm of the anterior tibial artery is rare, it should be kept in mind when a patient presents with abnormal swelling and pain after ankle arthroscopy. When a prompt diagnosis of pseudoaneurysm is made, referral to a vascular surgery team for prompt treatment is warranted.

In conclusion, we reported a rare case of a 19-year-old soccer player who presented with pseudoaneurysm of the anterior tibial artery following ankle arthroscopy and underwent anastomosis for the anterior tibial artery injury. Ankle arthroscopy carries a lower risk of vascular complications when standard anterolateral and anteromedial portals are used. However, the thickness of the fat pad at the anterior ankle affords little protection for the thin-walled anterior tibial artery, rendering it susceptible to indirect damage during procedures performed on the anterior ankle joint. Excessive distraction of the ankle puts the neurovascular structures at greater risk for iatrogenic injury of the anterior tibial artery during ankle arthroscopy [35]. Surgeons as well as other health professionals, such as nurses and physiotherapists, should look carefully for postoperative ankle swelling and pain after ankle arthroscopy because a prompt diagnosis is essential to the appropriate and successful management of pseudoaneurysm of the anterior tibial artery.

## Figures and Tables

**Table 1:** Cases of pseudoaneurysm of the anterior tibial artery after ankle arthroscopy reported in the literature.

Author, year	Number of cases	Age (y.o.)	Sex	Comorbidity	Procedures during ankle arthroscopy	Time to surgery for ATA	Size of pseudoaneurysm	Treatment for ATA pseudoaneurysm
O'Farrell et al. 1997 [10]	1	30	Male	Prosthetic aortic valve implantation	Removal of anterior tibiotalar osteophytes	1 week	2.0 × 2.0 cm	Ligation/anastomosis
Salgado et al. 1998 [11]	1	12	Female	None	Diagnostic arthroscopy	2 months	2.0 × 2.5 cm	Ligation
Mariani et al. 2001 [12]	1	50	Female	None	Synovectomy	1 week	2.0 × 2.5 cm	Ligation/vein graft
Darwish et al. 2004 [13]	1	70	Female	None	Synovectomy	6 weeks	4.0 × 4.0 cm	Ligation
Kotwal et al. 2007 [14]	1	20	Male	Hemophilia A	Excision of tibial osteophyte, debridement of the ankle	10 days	2.8 × 1.7 cm	Ligation/vein graft
Jang et al. 2008 [15]	1	25	Male	None	Synovectomy, ATFL reconstruction	8 weeks	3.5 × 2.8 × 1.9 cm	Compression
Ramavath et al. 2009 [16]	1	39	Female	Rheumatoid arthritis	Synovectomy	3 weeks	3.0 × 6.0 cm	Ligation
Brimmo and Parekh 2010 [17]	1	36	Male	None	Synovectomy, microfracture for OCL	11 weeks	NA	Embolisation
Jacobs et al. 2011 [18]	1	63	Female	Atrial fibrillation	Synovectomy	Approximately 12 days	NA	Embolisation
Jeffery et al. 2014 [19]	1	80	Male	Gout	NA	32 days	3.2 cm	Ligation
Chamseddin and Kirkwood 2016 [20]	1	35	Male	Hemophilia A	Debridement of anterior tibiotalar exostosis, synovectomy	Approximately 49 days	3.5 × 6.0 × 3.2 cm	Ligation
This case	1	19	Male	None	Debridement of anterior tibiotalar osteophyte, synovectomy	19 days	2.5 × 2.2 × 1.3 cm	Anastomosis

ATA: anterior tibial artery; ATFL: anterior tibiofibular ligament; NA: not available; OCL: osteochondral lesion.
